# The *Drosophila* Forkhead/Fox transcription factor Jumeau mediates specific cardiac progenitor cell divisions by regulating expression of the kinesin Nebbish

**DOI:** 10.1038/s41598-021-81894-1

**Published:** 2021-02-05

**Authors:** Andrew J. Kump, Manoj Panta, Kristopher R. Schwab, Mark H. Inlow, Shaad M. Ahmad

**Affiliations:** 1 Department of Biology, Indiana State University, Terre Haute, IN 47809 USA; 2 The Center for Genomic Advocacy, Indiana State University, Terre Haute, IN 47809 USA; 3 Rich and Robin Porter Cancer Research Center, Indiana State University, Terre Haute, IN 47809 USA; 4 Department of Mathematics and Computer Science, Indiana State University, Terre Haute, IN 47809 USA

**Keywords:** Genetics, Development, Drosophila, Developmental biology, Musculoskeletal development, Cell division

## Abstract

Forkhead (Fkh/Fox) domain transcription factors (TFs) mediate multiple cardiogenic processes in both mammals and *Drosophila*. We showed previously that the *Drosophila* Fox gene *jumeau* (*jumu*) controls three categories of cardiac progenitor cell division—asymmetric, symmetric, and cell division at an earlier stage—by regulating Polo kinase activity, and mediates the latter two categories in concert with the TF Myb. Those observations raised the question of whether other *jumu*-regulated genes also mediate all three categories of cardiac progenitor cell division or a subset thereof. By comparing microarray-based expression profiles of wild-type and *jumu* loss-of-function mesodermal cells, we identified *nebbish* (*neb*), a kinesin-encoding gene activated by *jumu*. Phenotypic analysis shows that *neb* is required for only two categories of *jumu*-regulated cardiac progenitor cell division: symmetric and cell division at an earlier stage. Synergistic genetic interactions between *neb*, *jumu*, *Myb*, and *polo* and the rescue of *jumu* mutations by ectopic cardiac mesoderm-specific expression of *neb* demonstrate that *neb* is an integral component of a *jumu*-regulated subnetwork mediating cardiac progenitor cell divisions. Our results emphasize the central role of Fox TFs in cardiogenesis and illustrate how a single TF can utilize different combinations of other regulators and downstream effectors to control distinct developmental processes.

## Introduction

While at least eight Forkhead (Fkh/Fox) domain transcription factors (TFs) are required for proper cardiac development in mammals^1–16^, and mutations in four Fox genes have been linked to human congenital heart defects^17–24^, relatively little is known about the molecular mechanisms or the downstream target genes by which these Fox TF-mediated developmental functions are brought about^25^. Our previous work had identified the conserved cardiogenic roles of two Fox genes, *jumeau* (*jumu*) and *Checkpoint suppressor homologue* (*CHES-1-like*) in both the specification and subsequent division of progenitor cells in the *Drosophila* heart^26–28^.

We showed previously that both *jumu* and *CHES-1-like* function in a mutually redundant manner to transcriptionally activate the expression of *heartless* (*htl*) and *frizzled* (*fz*), which respectively encode the receptors in a FGF-signaling pathway and a Wnt-signaling pathway required for cardiac progenitor specification; consequently, lacking both Fox genes results in a failure of much of the heart to be specified^26^. We also demonstrated that *jumu* and *CHES-1-like* mediate three distinct categories of cell division that generate the correct subtypes and numbers of cells constituting the heart—asymmetric cardiac progenitor cell divisions, symmetric cardiac progenitor cell divisions, and cell divisions at an earlier stage that produced the cardiac precursor cells—by regulating the activity of the conserved kinase Polo^28^. In addition, our investigations revealed that the TF Myb acts synergistically with Jumu, CHES-1-like, and Polo to mediate only two of these three categories of cardiac progenitor cell division: symmetric and cell division at an earlier stage^27^. In contrast, asymmetric cardiac progenitor cell division involved Jumu and CHES-1-like utilizing Polo to phosphorylate Partner of numb (Pon) and thereby bring about the localization of membrane-associated Numb protein to one pole of the dividing cell^28,29^.

Additionally, we found that Fox TF binding sites were significantly enriched in the enhancers of genes expressed in the heart^27,30^, and that loss-of-function mutations in *jumu* or *CHES-1-like* significantly altered the expression levels of many known cardiac genes^26^. Taken together, these data indicate that the Fox TFs regulate a large number of genes in addition to *polo*, *htl*, and *fz* in order to mediate cardiogenesis.

Collectively, these observations raise three intriguing developmental questions. First, are additional Fox TF-regulated genes besides *polo*, *htl*, and *fz* involved in the two distinct cardiogenic processes—cardiac progenitor specification and cardiac progenitor cell division—that we have discovered as being mediated by the Fox TFs to date?

Second, *jumu* and *CHES-1-like* were both seen to be simultaneously regulating the activity of each relevant downstream target gene (*htl*, *fz*, or *polo*) whenever any one of these target genes was characterized as mediating a cardiogenic process. Thus, a particularly germane question is whether there are any downstream cardiogenic targets and, by extension, associated pathways or subnetworks, that are exclusively regulated and mediated by one, but not the other, of these two Fox TFs.

Third, while *Myb* itself acts in concert with both Fox genes at the same hierarchical level to mediate only two of the three categories of cardiac progenitor cell divisions, *polo*, the downstream target of both *jumu* and *CHES-1-like*, is utilized for all three. Thus, another pertinent question is whether every Fox TF-regulated gene utilized in cardiac progenitor cell division also plays critical roles in all three categories of cell division or is necessary for only certain specific subsets.

Here we show that the kinesin-encoding gene *nebbish* (*neb*) is activated by only one Fox gene, *jumu*; that *neb* is required in conjunction with *polo* and *Myb* to mediate cardiac progenitor cell divisions; and that *neb* is necessary for only two of the three categories of cardiac progenitor cell division—symmetric and cell division at an earlier stage—thereby extending our understanding of the Fox-regulated subnetworks mediating cardiogenesis.

## Results

### *jumu*, but not *CHES-1-like*, activates mesodermal expression of *neb*

One of our goals was to discover downstream genes mediating cardiogenic processes, if present, that were regulated by one, but not the other, of the twin Fox TFs Jumu and CHES-1-like. We had previously used flow cytometry and Affymetrix microarrays to obtain genome-wide transcription expression profiles of purified mesodermal cells from wild-type embryos, embryos homozygous for a null mutation in *jumu*, and embryos homozygous for a null mutation in *CHES-1-like* at embryonic stages 11 and early 12, i.e. when cardiac progenitor specification and cell division occur in *Drosophila*^26^. Reasoning that cardiogenic genes regulated by a Fox TF are likely to exhibit differential expression between the heart-producing mesoderm in wild-type and Fox loss-of-function mutant embryos, we compared the transcription expression profiles between mesodermal cells from wild-type embryos and the Fox gene mutants. We found that *neb* had its expression levels significantly downregulated in *jumu* null mutants [log_2_(fold change) =  − 0.69058; adjusted *P*-value = 1.65 × 10^−5^ after correction for multiple hypothesis testing] but remained essentially unaffected in *CHES-1-like* null mutants [log_2_(fold change) = 0.14551; adjusted *P*-value = 0.195].

**Figure 1:**
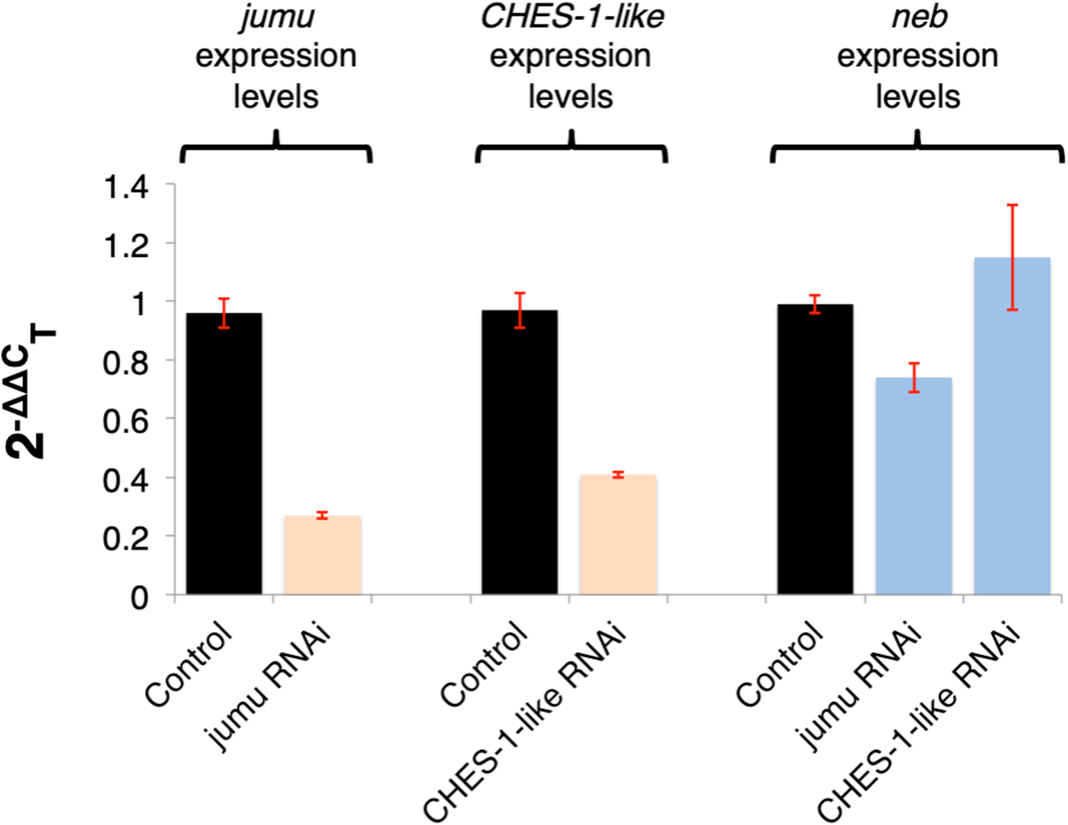
*jumu*, but not *CHES-1-like*, activates mesodermal expression of *neb*. Relative *jumu*, *CHES-1-like*, and *neb* gene expression levels in *jumu* or *CHES-1-like* RNAi knockdown embryos assessed and compared to control embryos via RT-qPCR and the 2^−ΔΔC^_T_ method. Error bars indicate standard deviations. Mesoderm-targeted *jumu* and *CHES-1-like* RNAi do indeed reduce *jumu* and *CHES-1-like* gene expression levels compared to controls, respectively, indicating that these RNAi constructs result in the relevant Fox gene knockdowns. However, while mesoderm-targeted *jumu* RNAi knockdown also decreases *neb* transcript expression levels relative to the control, no such reduction is detected in mesoderm-targeted *CHES-1-like* knockdowns.

In order to independently verify the effects of these two Fox genes on *neb* expression, we used the pan-mesodermal *twi-GAL4* driver to knock down either *jumu* or *CHES-1-like* via RNA interference (RNAi) exclusively in the mesoderm. We then assessed and compared the resulting *neb* transcript levels at stages 11–12 with that in control embryos by reverse transcription quantitative real-time PCR (RT-qPCR). Since *jumu* and *CHES-1-like* are also expressed in tissues other than the mesoderm^31^, null mutations in the Fox genes could potentially also affect *neb* expression levels in these non-mesodermal tissues. Utilizing exclusively mesoderm-targeted RNAi knockdowns of these Fox genes instead of null mutations in *jumu* or *CHES-1-like* thus ensured that any detected alterations in *neb* transcript levels in total RNA were due solely to the effects of the Fox genes in the mesoderm. Our RT-qPCR assays revealed a decrease in *neb* expression levels in mesoderm-targeted *jumu* knockdowns compared to that in control embryos containing only the *twi-GAL4* driver, but detected no such reduction in mesoderm-targeted *CHES-1-like* knockdowns (Fig. 1).

Collectively, both our microarray-based expression profiling data and RT-qPCR-mediated assessment of Fox gene knockdowns indicated that *jumu*, but not *CHES-1-like*, regulates *neb* in the mesoderm by activating its expression.

### *neb* is expressed in the cardiac mesoderm during cardiac progenitor specification and cell division

**Figure 2:**
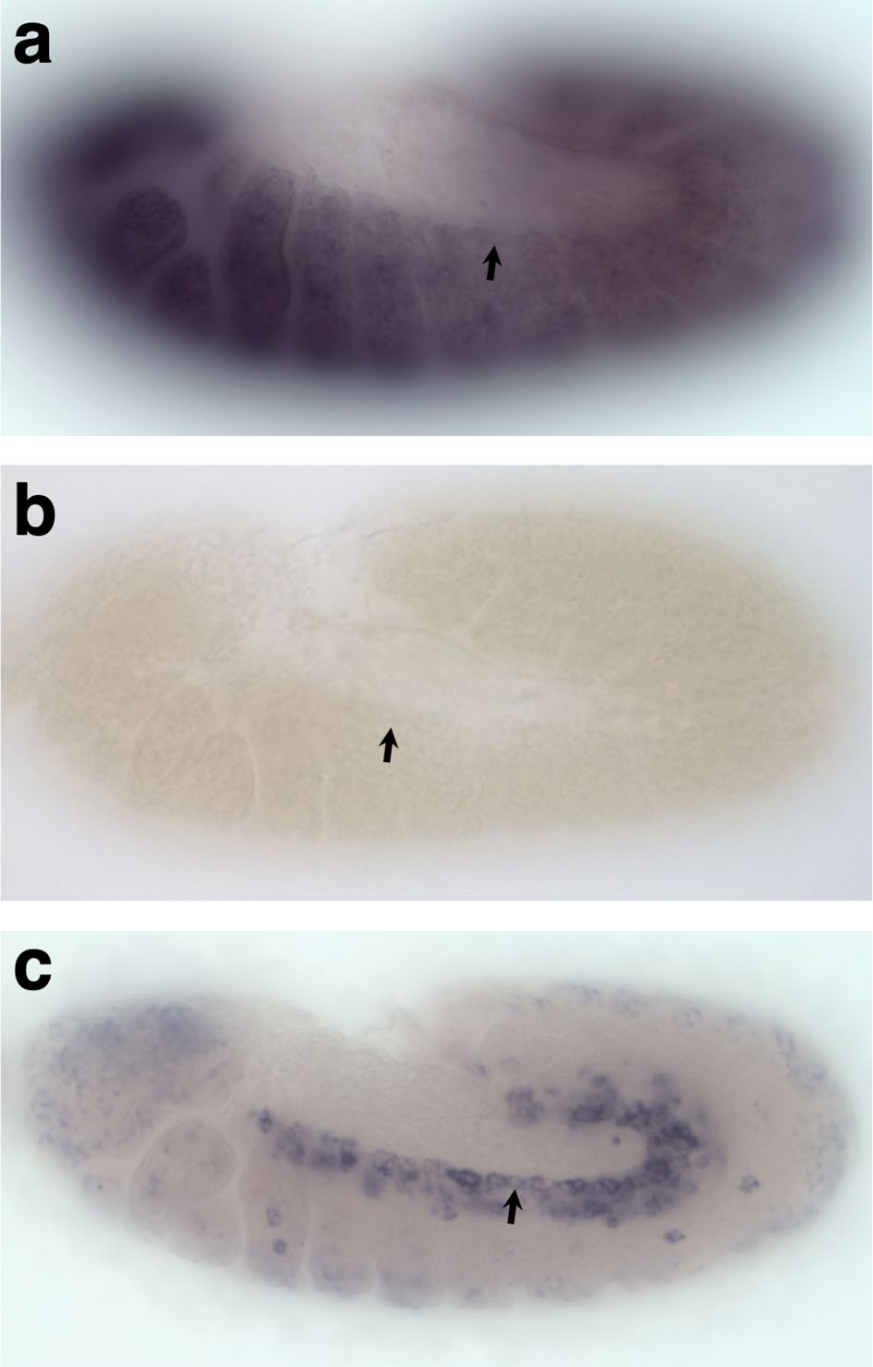
*neb* mRNA expression in the cardiac mesoderm. (**a**) Antisense riboprobe hybridizing to the mRNA shows that the *neb* transcript is expressed in the cardiac mesoderm in stage 11 wild-type embryos. (**b**) Control in situ hybridization with a sense riboprobe complementary to that used in (**a**) developed under identical conditions for the same period. The absence of similar staining in (**b**) indicates that the staining in (**a**) is *neb*-specific and not an artifact. (**c**) In situ hybridization of stage 11 embryos of the genotype *TinD-GAL4/+*; *jumu*^*Df(3R)Exel6157*^ *svp-lacZ/jumu*^*Df(3R)Exel6157*^ *UAS-neb* with the same antisense *neb* riboprobe as in (**a**) developed using identical conditions but for a much shorter period. The specific *TinD-GAL4*-targeted expression of *neb* to the cardiac mesoderm is clearly observed while the staining for endogenous *neb* expression is considerably fainter due to the shorter period used. The arrows indicate cells of the cardiac mesoderm in all three panels (**a**–**c**).

*neb* had previously been described as being expressed in the nervous tissue during mid-embryogenesis^31^. Using in situ hybridization, we found that the *neb* transcript is also expressed, albeit at lower levels, in the cardiac mesoderm and in subsets of the somatic mesoderm at embryonic stages 11 and 12, i.e. when the cardiac progenitor cells are specified and undergo cell division (Fig. 2a,b). However, the expression pattern of the transcript made it difficult to determine unequivocally whether the expression level of *neb* is lower specifically in the cardiac mesoderm of *jumu* loss-of-function mutants by in situ hybridization assays. Regardless, the observations that *neb* is both expressed in the cardiac mesoderm and is transcriptionally activated mesodermally by *jumu*, collectively raise the question of whether *neb* has a critical a role in cardiogenesis.

### *neb* is required in the cardiac mesoderm for two distinct categories of cardiac progenitor cell divisions

**Figure 3:**
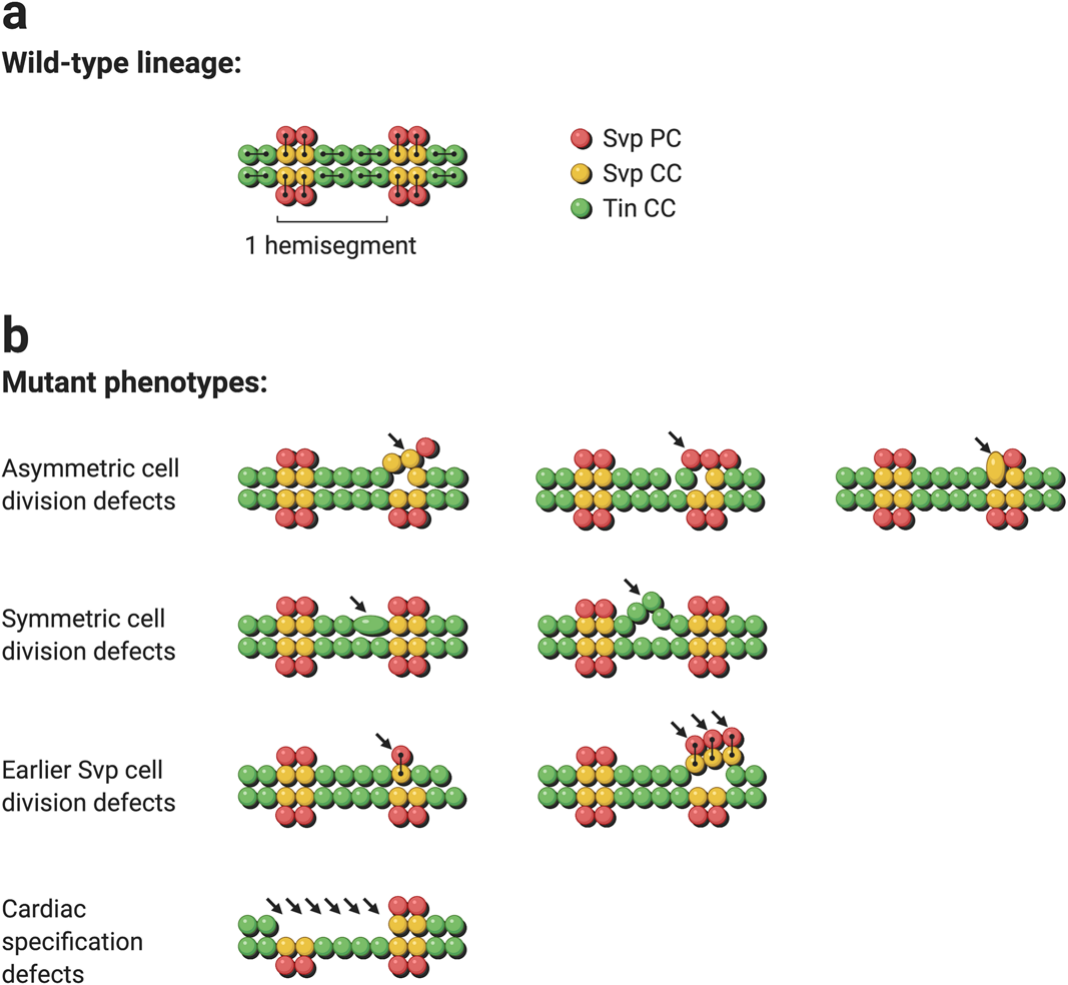
Schematic showing cell lineage relationships in a wild-type *Drosophila* embryonic heart and the expected phenotypes due to defects in cardiac progenitor specification or cell division. (**a**) Lines connect daughter cells arising from the division of each precursor cell. (**b**) Defects in cardiac progenitor cell specification would be expected to result in the absence of all the cardial cells in one or more hemisegments. Defects in asymmetric cell division would result in an increase or reduction in the number of Svp-CCs accompanied by a corresponding decrease or increase in the number of Svp-PCs, or larger Svp-CC nuclei with missing corresponding Svp-PCs due to errors in karyokinesis. Defective symmetric cell division would result in an alteration in the expected number of four Tin-CCs per hemisegment, while errors at the earlier stage of cell division producing the two Svp precursors would result in hemisegments with either one or three pairs of Svp-CCs and Svp-PCs instead of the customary two pairs.

The metamerically repeated and stereotyped pattern of distinct cardiac cell types in the embryonic *Drosophila* heart makes it particularly amenable to discovering whether *neb* has one or more crucial cardiogenic roles, and what those roles might be, by examining the phenotypes of *neb* loss-of-function mutations. The heart itself is comprised of two major cell types: an inner tube of *Myocyte enhancer factor 2* (*Mef-2*)-expressing contractile cardial cells (CCs) and an outer sheath of *Zn finger homeodomain 1* (*zfh1*)-expressing nephrocytic pericardial cells. From anterior to posterior, the cardial cells consist of two Seven up-expressing CCs (Svp-CCs) followed by four Tinman-expressing CCs (Tin-CCs) in every repeating hemisegment. These cardial cells are surrounded by a larger number of pericardial cells, which includes two Seven up-expressing PCs (Svp-PCs) that are located laterally to the CCs in each and every hemisegment. The different cellular subtypes comprising these cardial and pericardial cells arise through a stereotyped series of cardiac progenitor cell divisions^32–35^. Specifically, cell division at an earlier stage ensures that there are two Svp precursor cells per hemisegment. Subsequently, the two Svp-CCs in wild-type cardiac hemisegments arise by two asymmetric progenitor cell divisions from these two Svp precursor cells, with each cell division generating one Svp-CC and one Svp-PC (Fig. 3a, yellow and red cells, respectively). In contrast, two symmetric cell divisions give rise to the four Tin-CCs (Fig. 3a, green cells) per hemisegment from two other precursor cells.

**Figure 4:**
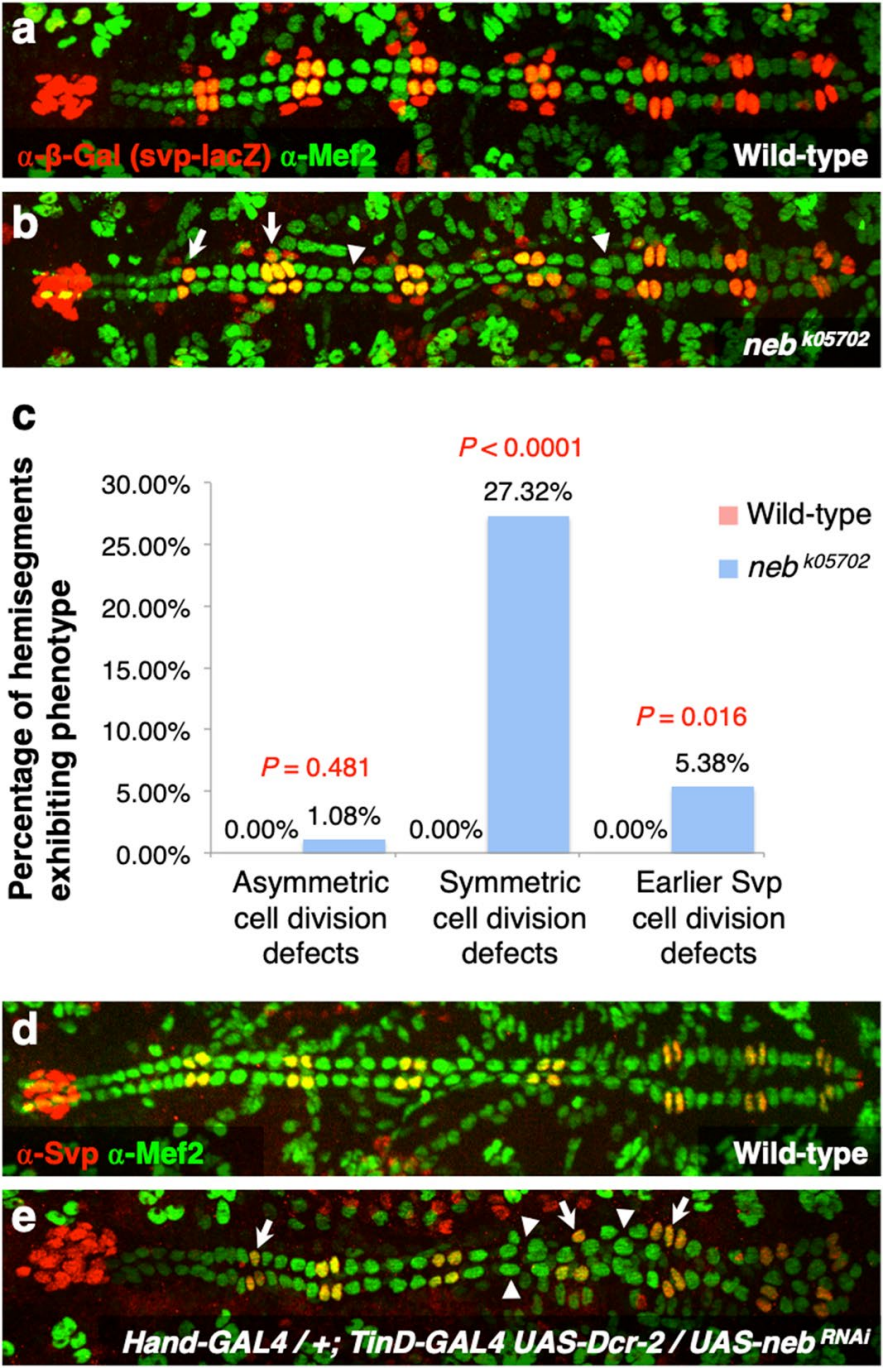
Cardiac progenitor cell division defects associated with *neb* loss of function. (**a**) A heart from an otherwise wild-type embryo bearing one copy of the *svp-lacZ* enhancer trap showing hemisegments consisting of four Tin-CCs (green), two Svp-CCs (yellow), and two Svp-PCs (red). (**b**) A heart from an embryo homozygous for the *neb*^*k05702*^ null mutation (and also carrying one copy of the *svp-lacZ* enhancer trap) exhibiting both cardiac progenitor symmetric cell division defects (arrowheads) and defects at an earlier round of cell division specifying the number of Svp precursors (arrows). In these images (derived by flattening z-stacks), a few Svp-PCs are hidden underneath the CCs or appear quite faint in certain hemisegments, but all were clearly discernible in the individual planes of the z-stacks from which the images were obtained. (**c**) Percentage of hemisegments exhibiting each type of cardiac progenitor cell division defect in embryos that are wild-type (n = 196 hemisegments) or homozygous for the *neb*^*k05702*^ mutation (n = 186 hemisegments). The significance of each type of cell division defect in the *neb* mutants compared with wild-type is shown. (**d**) A wild-type heart stained to show only Tin-CCs (green) and Svp-CCs (yellow). Each hemisegment contains four Tin-CCs and two Svp-CCs. (**e**) Similar staining in embryos where *neb* has been knocked down specifically in the cardiac mesoderm exhibits changes in Tin-CC numbers and Svp-CC numbers consistent with symmetric (arrowheads) and earlier (arrows) cell division defects, respectively.

These lineage relationships allow us to use the numbers of the Tin-CCs, Svp-CCs, and Svp-PCs in individual hemisegments in *neb* loss-of-function mutants to determine whether *neb* is mediating one or more of these three known categories of cardiac progenitor cell divisions (Fig. 3b). If *neb* mutations cause asymmetric cell division defects, we would expect to see an increase or reduction in the number of Svp-CCs accompanied by a corresponding decrease or increase in the number of Svp-PCs, or larger Svp-CC nuclei with missing corresponding Svp-PCs due to errors in karyokinesis. Defective symmetric cell division, on the other hand, would be identified as any alteration in the expected number of four Tin-CCs per hemisegment, while errors at the earlier stage of cell division producing the two Svp precursors would result in hemisegments with either one or three pairs of Svp-CCs and Svp-PCs instead of the customary two pairs^28^. Our examination found that embryos homozygous for the *neb*^*k05702*^ loss-of-function allele exhibited a significant increase over wild-type both in the fraction of hemisegments with excess or fewer Tin-CCs (*P* = 2.00 × 10^−6^), corresponding to symmetric cell division defects, and in the fraction of hemisegments with either one or three pairs of Svp-CCs and Svp-PCs (*P* = 0.016), corresponding to earlier cell division defects affecting the number of Svp precursors (Fig. 4a–c, Supplementary Table S1). The fraction of hemisegments exhibiting asymmetric cell division defects, in contrast, was miniscule and not significantly different (*P* = 0.481) from that in wild-type embryos (Fig. 4a–c, Supplementary Table S1). Collectively, our results demonstrate that *neb* is necessary to mediate both symmetric cell divisions and the earlier round of cell divisions that give rise to the Svp precursors, but is not required for asymmetric cell divisions. Of note, we did not detect any instance where all the cardial cells in one or more entire hemisegments were missing, as would be expected in the case of cardiac progenitor specification defects, and were seen when *htl* or *fz* functions were disrupted^26, 36–38^.

We next attempted to ascertain whether this *neb* function, necessary for mediating both symmetric cell divisions and divisions at the earlier stage affecting the number of Svp precursor cells, is required specifically in the cardiac progenitors. We thus knocked down *neb* activity specifically in the cardiac progenitors by cardiac mesoderm and heart-targeted RNA interference (RNAi) directed by the *TinD-GAL4*^39^ and *Hand-GAL4*^40^ drivers and examined the resulting embryos for alterations in expected cardial cell numbers consistent with errors in cardiac progenitor cell divisions. Staining with appropriate antibodies showed hemisegments with localized changes in the number of Tin-CCs corresponding to symmetric cell division defects and Svp-CCs corresponding to defective cell division at an earlier stage (Fig. 4d,e, Supplementary Fig. S1, Supplementary Table S2).

Taken together, these results demonstrate that the requirement of *neb* for mediating two distinct categories of cardiac progenitor cell division is autonomous to the cardiac mesoderm and raise the question of which cell division pathway or subnetwork *neb* is involved in, one that we address in the following experiments.

### Synergistic genetic interactions between *neb*, *jumu*, *polo*, and *Myb*

We had previously shown that *jumu* and *CHES-1-like* regulate *polo* to mediate all three categories of cardiac progenitor cell divisions^28^; and that *Myb* functions synergistically with *jumu*, *CHES-1-like*, and *polo* to control both symmetric and earlier cardiac progenitor cell divisions^27^, the same two cardiogenic processes mediated by *neb*. These observations raise the questions of whether *neb* functions through the same genetic pathways as *Myb*, *jumu*, and *polo*, and, if it does, for which specific categories of cardiac progenitor cell divisions. Operating on the premise that we may expect to see synergistic, i.e. more than merely additive, genetic interactions between mutant alleles of *neb* and another gene if they both function together in the same genetic pathway, we quantified and compared the phenotypes of single heterozygotes of mutations in *neb* and single heterozygotes of mutations in *jumu*, *polo*, or *Myb*, with those of embryos that were doubly heterozygous for mutations in both *neb* and *jumu*, *neb* and *polo*, and *neb* and *Myb*, respectively.

**Figure 5:**
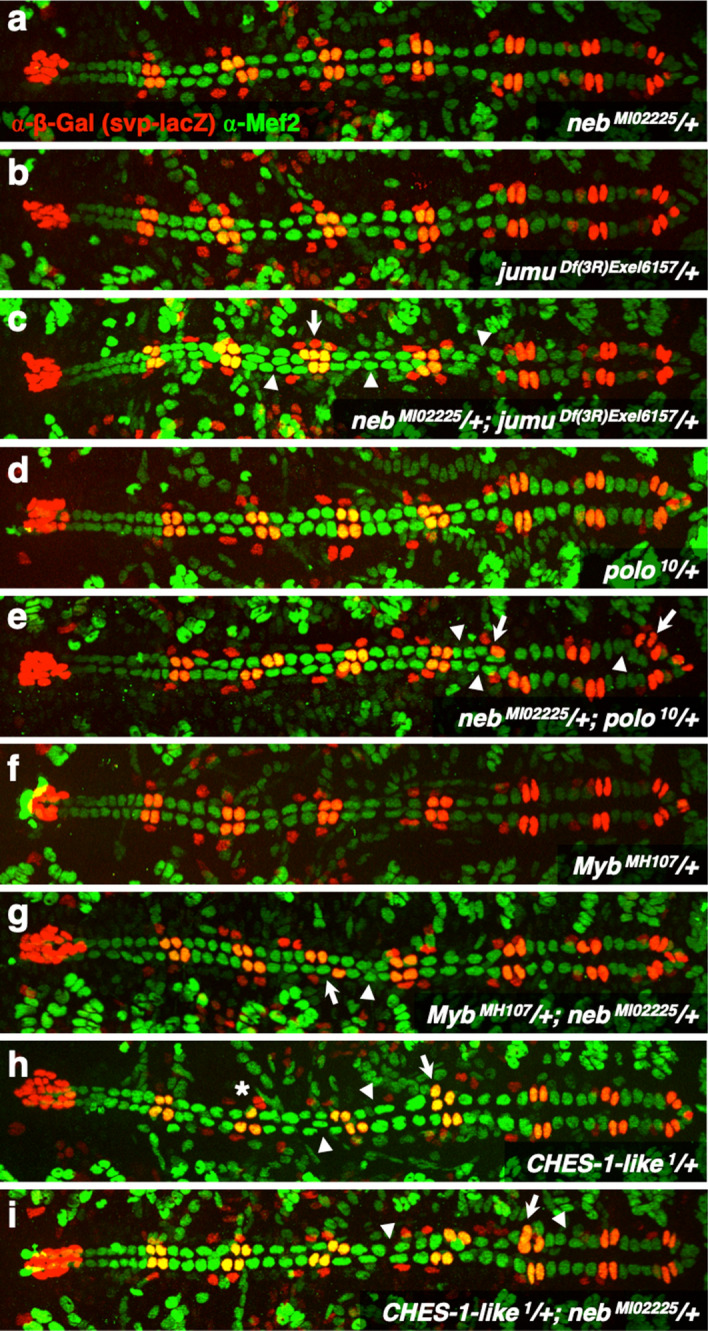
*neb* exhibits synergistic genetic interactions with *jumu*, *polo*, and *Myb*, but not with *CHES-1-like*. Representative hearts from embryos (**a**) heterozygous for the *neb*^*MI02225*^ allele, (**b**) heterozygous for the *jumu*^*Df(3R)Exel6157*^ allele, (**c**) doubly heterozygous for both the *neb*^*MI02225*^ and *jumu*^*Df(3R)Exel6157*^ alleles, (**d**) heterozygous for the *polo*^*10*^ allele, (**e**) doubly heterozygous for both the *neb*^*MI02225*^ and *polo*^*10*^ alleles, (**F**) heterozygous for the *Myb*^*MH107*^ allele, (**g**) doubly heterozygous for both the *neb*^*MI02225*^ and *Myb*^*MH107*^ alleles, (**h**) heterozygous for the *CHES-1-like*^*1*^ allele, and (**i**) doubly heterozygous for both the *neb*^*MI02225*^ and *CHES-1-like*^*1*^ alleles. All of these embryos carry one copy of the *svp-lacZ* enhancer trap, thereby allowing the identification of Tin-CCs (green), Svp-CCs (yellow), and Svp-PCs (red). In these images (derived by flattening z-stacks), a few Svp-PCs are hidden underneath the CCs or appear quite faint in certain hemisegments, but all were clearly discernible in the individual planes of the z-stacks from which the images were obtained. Asymmetric cell division defects are denoted by asterisks, symmetric cell division defects by arrowheads, and earlier Svp precursor-determining cell division defects by arrows.

**Figure 6:**
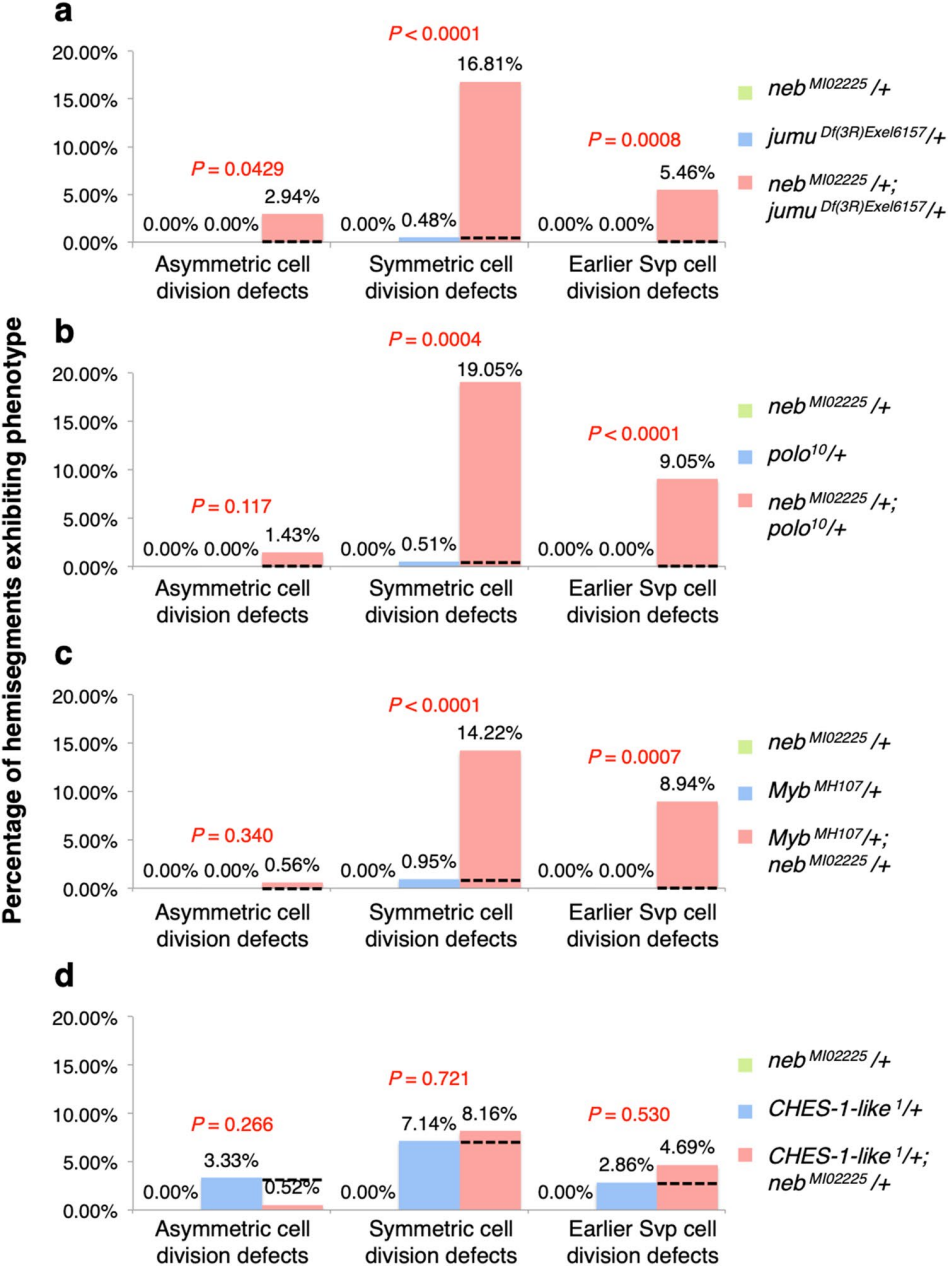
Quantification and significance of cardiac progenitor cell division defects in genetic interaction assays. (**a**) Percentage of hemisegments exhibiting each type of cardiac progenitor cell division defect in embryos that are heterozygous for the *neb*^*MI02225*^ allele (n = 188 hemisegments), heterozygous for the *jumu*^*Df(3R)Exel6157*^ allele (n = 210 hemisegments), or doubly heterozygous for both the *neb*^*MI02225*^ and *jumu*^*Df(3R)Exel6157*^ alleles (n = 238 hemisegments). (**b**) Percentage of hemisegments exhibiting each type of cardiac progenitor cell division defect in embryos that are heterozygous for the *neb*^*MI02225*^ allele, heterozygous for the *polo*^*10*^ allele (n = 196 hemisegments), or doubly heterozygous for both the *neb*^*MI02225*^ and *polo*^*10*^ alleles (n = 210 hemisegments). (**c**) Percentage of hemisegments exhibiting each type of cardiac progenitor cell division defect in embryos that are heterozygous for the *neb*^*MI02225*^ allele, heterozygous for the *Myb*^*MH107*^ allele (n = 193 hemisegments), or doubly heterozygous for both the *neb*^*MI02225*^ and *Myb*^*MH107*^ alleles (n = 179 hemisegments). (**d**) Percentage of hemisegments exhibiting each type of cardiac progenitor cell division defect in embryos that are heterozygous for the *neb*^*MI02225*^ allele, heterozygous for the *CHES-1-like*^*1*^ allele (n = 210 hemisegments), or doubly heterozygous for both the *neb*^*MI02225*^ and *CHES-1-like*^*1*^ alleles (n = 192 hemisegments).

Double heterozygotes for both *neb* and *jumu* mutations exhibit symmetric cell division defects and earlier Svp precursor-determining cell division defects at frequencies that are significantly more severe (*P* = 1.80 × 10^−5^ and *P* = 8.23 × 10^−4^, respectively) than the additive effects of both the *neb* single heterozygotes and the *jumu* single heterozygotes (Figs. 5a–c, 6a, Supplementary Table S1). However, the increase in the frequency of asymmetric cell division defects in *neb*^*MI0225*^*/*+;* jumu*^*Df(3R)Exel6157*^*/*+double heterozygotes over the additive sum of those in the component *neb*^*MI0225*^*/*+and *jumu*^*Df(3R)Exel6157*^*/*+single heterozygotes is barely significant (*P* = 0.0429) (Figs. 5a–c, 6a, Supplementary Table S1). Our results thus indicate clearly that *jumu* and *neb* do indeed mediate both symmetric and earlier Svp precursor-determining cardiac progenitor cell divisions through the same genetic pathways, but the evidence for *neb* functioning in concert with *jumu* to bring about asymmetric cardiac progenitor cell divisions as well is not as strong. Note that these genetic interaction results are also consistent with our initial finding of *neb* being required for symmetric and earlier, but not asymmetric, cardiac progenitor cell divisions.

Since *jumu* brings about all three categories of cardiac progenitor cell divisions by regulating the activity of Polo kinase^28^, we used similar genetic interaction assays next to assess whether *neb* and *polo* also function through the same genetic pathways. Our results revealed highly significant synergistic genetic interactions between *neb* and *polo* for both symmetric cell divisions and earlier cell divisions (*P* = 3.89 × 10^−4^ and *P* = 1.00 × 10^−6^, respectively), but no significant interactions in the case of asymmetric cardiac progenitor cell divisions (*P* = 0.117) (Figs. 5a,d,e, 6b, Supplementary Table S1).

Since *Myb* has been shown to regulate *polo* expression^41^, and act synergistically with *jumu* to mediate only symmetric and earlier Svp precursor-determining cardiac progenitor cell divisions^27^, we next addressed whether *neb* works in concert with *Myb* via genetic interaction assays, and in which classes of cardiac progenitor cell divisions. Significant synergistic genetic interactions between *neb* and *Myb* were also detected in the case of both symmetric cell divisions and earlier cell divisions (*P* = 7.60 × 10^−5^ and *P* = 7.43 × 10^−4^, respectively), but not for asymmetric cell divisions (*P* = 0.340) (Figs. 5a,f,g, 6c, Supplementary Table S1).

Taken together, these results and our initial findings show that *neb* is necessary for only two of the three categories of cardiac progenitor cell divisions, symmetric and earlier, and that it mediates these two types of cell divisions by working in concert with *jumu*, *Myb*, and *polo* through the same genetic pathways.

### Similar synergistic genetic interactions are not detected between *neb* and *CHES-1-like*

As described previously, both of the *jumu*-regulated cardiogenic processes that we had discovered, cardiac progenitor specification and cell division, are also regulated by another Fox TF, CHES-1-like. Furthermore, the downstream genes used for each of these processes, *htl* and *fz* in the case of cadiac mesoderm specification, and *polo* in the case of cardiac progenitor cell divisions, are each also regulated by both *jumu* and *CHES-1-like*^27,28^. *neb*, however, provides an exception to this trend because, based on our expression profiling data, it is transcriptionally regulated only by *jumu*, and not by *CHES-1-like*. Does the observation that *CHES-1-like* does not regulate *neb* imply that *neb* functions completely independently of *CHES-1-like*, or are they both components of the same genetic subnetwork?

In an attempt to answer this question, we assessed potential genetic interactions between *neb* and *CHES-1-like* in mediating cardiac progenitor cell divisions, based on the premise that such interactions are likely to be synergistic if *neb* and *CHES-1-like* function in concert in the same pathways. The frequency of cardiac progenitor cell division defects in *CHES-1-like*^*1*^*/*+;* neb*^*MI0225*^*/*+ double heterozygotes was not significantly different from the additive sum of those of the *CHES-1-like*^*1*^*/*+ and *neb*^*MI0225*^*/*+ single heterozygotes during asymmetric (*P* = 0.266), symmetric (*P* = 0.721), or earlier Svp precursor-determining cell divisions (*P* = 0.530) (Figs. 5a,h,i, 6d, Supplementary Table S1). The lack of synergistic genetic interactions between *neb* and *CHES-1-like* in any of the three cardiac progenitor cell division categories suggests strongly that these two genes function independently of each other in these cardiogenic processes.

### Cardiac mesoderm-targeted ectopic expression of *neb* partially rescues both symmetric and earlier cardiac progenitor cell division defects in embryos lacking *jumu* function

Our results so far show that *jumu* activates *neb* and that *neb* is specifically required in the cardiac mesoderm for mediating symmetric and earlier Svp precursor-determining cell divisions, i.e. two of the three categories of *jumu*-regulated cardiac progenitor cell divisions. The most parsimonious hypothesis that can explain these observations is that *jumu* transcriptionally upregulates *neb* in the cardiac mesoderm to bring about symmetric and earlier cardiac progenitor cell divisions, and that in *jumu* loss-of-function mutations the ensuing reduction in *neb* expression levels in this tissue results in defects in only these two types of cell divisions. However, the nature of *neb* expression prevented us from qualitatively assessing via in situ hybridization assays whether the reduction of *neb* transcript levels in the entire mesoderm in *jumu* mutants reflected a reduction of *neb* levels specifically in the cardiac mesoderm.

**Figure 7:**
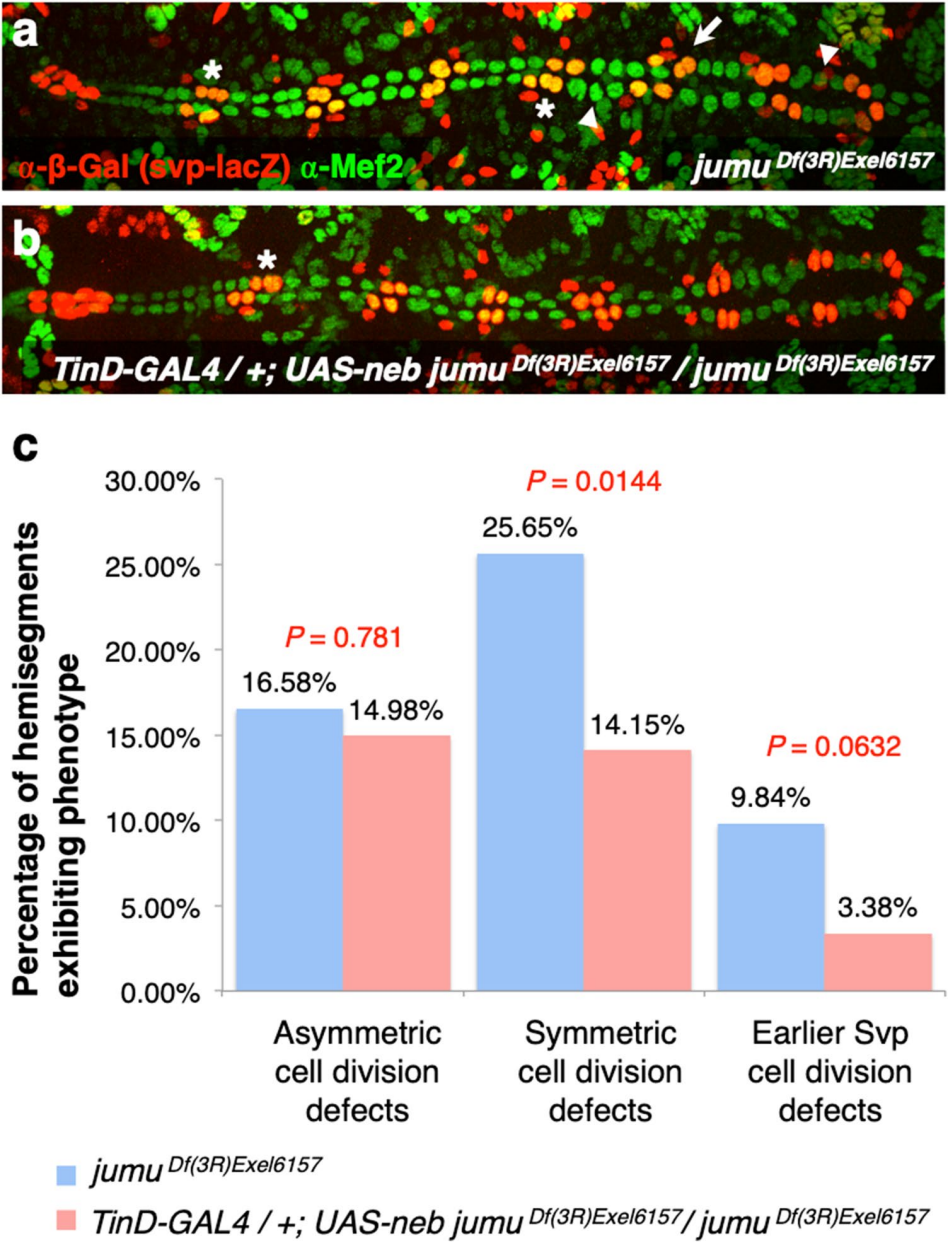
Cardiac mesoderm-targeted ectopic expression of *neb* partially rescues both symmetric and earlier Svp precursor-determining cardiac progenitor cell division defects in embryos lacking *jumu* function. (**a**) Representative heart from an embryo homozygous for the *jumu*^*Df(3R)Exel6157*^ null mutation exhibiting asymmetric cell division defects (asterisks), symmetric cell division defects (arrowheads), and earlier Svp precursor-determining cell division defects (arrow). (**b**) Representative heart from an embryo homozygous for the *jumu*^*Df(3R)Exel6157*^ null mutation but ectopically expressing *neb* in the cardiac mesoderm exhibiting only asymmetric cell division defects. All of these embryos possess one copy of the *svp-lacZ* enhancer trap, allowing the identification of Tin-CCs (green), Svp-CCs (yellow), and Svp-PCs (red). (**c**) Quantification and significance of each type of cardiac progenitor cell division defect (n = 193 hemisegments for embryos homozygous for the *jumu*^*Df(3R)Exel6157*^ null mutation alone, and n = 205 hemisegments for embryos homozygous for the *jumu*^*Df(3R)Exel6157*^ null mutation but ectopically expressing *neb* in the cardiac mesoderm).

Therefore, to test this hypothesis, we ectopically expressed full-length *neb* specifically in the cardiac mesoderm in *jumu* loss-of-function mutants under the control of the *TinD-GAL4* driver (Fig. 2c). If our hypothesis that *neb* acts downstream of *jumu* specifically in the cardiac mesoderm is correct, then the cardiac mesoderm-specific ectopic expression of *neb* should be able to partially restore *neb* levels and rescue the symmetric and earlier cardiac progenitor cell division defects in *jumu* mutants. We found that ectopic expression of *neb* throughout the cardiac mesoderm does indeed significantly reduce the severity of defects of *jumu* mutants in symmetric cardiac progenitor cell divisions (*P* = 0.0144). Defects in Svp precursor-determining cell divisions are also rescued, albeit with borderline significance (*P* = 0.0632). In contrast, no significant difference is detected in asymmetric cell division defects between *jumu* mutants alone and *jumu* mutants expressing full-length *neb* (*P* = 0.781) (Fig. 7a–c, Supplementary Table S1). Collectively, our results thus indicate a downstream requirement of *neb* in the cardiac mesoderm for correct symmetric and earlier cell divisions mediated by *jumu*.

## Discussion

Our previous work had identified the roles of *htl* and *fz* as Fox TF-regulated genes that mediate cardiac progenitor specification and *polo* as yet another Fox TF-regulated gene that mediates three distinct categories of cardiac progenitor cell division^26,28^. In this study, we identify another Fox TF-regulated downstream gene, *neb*, which also mediates cardiac progenitor cell divisions. The previously characterized Fox downstream targets *htl*, *fz*, and *polo* are each regulated by both *jumu* and *CHES-1-like* simultaneously. Our expression profiling results indicate that *neb*, in contrast, is transcriptionally regulated only by Jumu, and not by CHES-1-like. This independence from *CHES-1-like* regulatory control is also supported by *neb* exhibiting synergistic genetic interactions with *jumu* in cardiac progenitor cell division subnetworks, but not with *CHES-1-like*.

We showed further that it is the activation of *neb* by Jumu specifically in the cardiac mesoderm that is necessary for proper cardiac progenitor cell divisions. It is possible that Jumu may also activate *neb* expression at other mesodermal domains besides the cardiac mesoderm: our expression profiling was based on the entire mesoderm, and in situ hybridization assays showed *neb* expression in subsets of the somatic mesoderm in addition to the cardiac mesoderm. However, the twin observations that cardiac mesoderm-specific knockdown of *neb* partially phenocopies *jumu* loss-of-function cardiac progenitor cell division defects and that cardiac mesoderm-specific ectopic expression of *neb* partially rescues *jumu* loss-of-function cell division defects demonstrate clearly that *jumu* mediates these cardiac progenitor cell division defects by activating *neb* expression specifically in the cardiac mesoderm.

**Figure 8:**
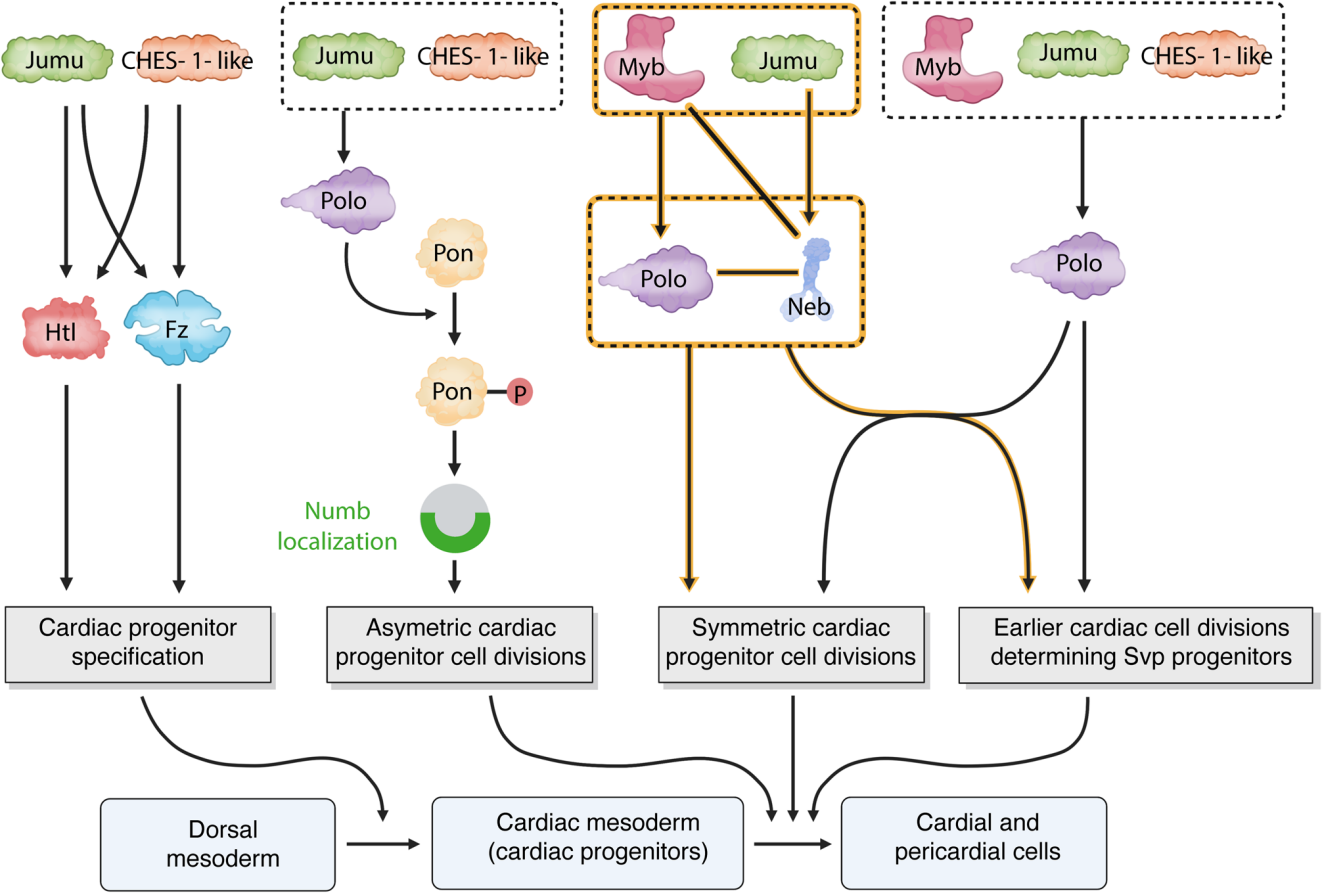
Model for *jumu* and *CHES-1-like*-regulated cardiogenic processes based on previous work and this current study. *jumu* and *CHES-1-like* initially transcriptionally activate *htl* and *fz* to mediate the specification of cardiac progenitors from the dorsal mesoderm by the FGF and Wnt-signaling pathways^26^. Polo, activated by the synergistic functions of *jumu* and *CHES-1-like*, then brings about the phosphorylation of Pon necessary for the localization of Numb protein during asymmetric cell division of the Svp precursor cells^28^. Additionally, *jumu*, *CHES-1-like*, and *Myb* also act synergistically to activate Polo to mediate both symmetric and earlier Svp precursor-determining cell divisions^27,28,41^. The results of the current study suggest the presence of an additional *jumu*-regulated pathway (highlighted in peach) that acts independently of *CHES-1-like*, involves *Myb*, *polo*, and *neb*, and mediates only symmetric and earlier Svp precursor-determining cardiac progenitor cell divisions. Lines without arrowheads connecting *neb* with *polo* and with *Myb* indicate that while these three genes do indeed function synergistically to mediate symmetric and earlier cardiac progenitor cell divisions, our data at present cannot determine whether *Myb* or *polo* are acting upstream or downstream of *neb*. Since both *polo* and *neb* are regulated by *jumu*, and since it is not yet known whether *polo* acts upstream or downstream of *neb*, these two genes have been drawn at the same level.

Our previous examinations of cardiac progenitor cell divisions had shown that while *jumu*, *CHES-1-like*, and *polo* are required for all three categories of cardiac progenitor cell division—asymmetric cell divisions, symmetric cell divisions, and cell divisions at an earlier stage that produce the Svp precursor cells—*Myb* is required for only the two latter classes^27,28^. In this study, we discovered that *neb*, like *Myb*, is also required for only the symmetric and earlier cardiac progenitor cell divisions, not asymmetric cell divisions. Furthermore, while *neb* exhibits no synergistic genetic interactions with *CHES-1-like* in any of the three classes of cardiac progenitor cell divisions, it displays synergistic genetic interactions with *Myb* and *polo* exclusively in symmetric and earlier Svp precursor-determining cell divisions. And while *neb* and *jumu* do show synergistic genetic interactions in all three categories of cardiac progenitor cell divisions, the interaction in the case of asymmetric cell divisions is barely significant. Finally, we find that ectopic cardiac mesoderm-specific expression of *neb* partially rescues symmetric and earlier cardiac progenitor cell division defects in *jumu* loss-of-function mutants, but not asymmetric cell division defects. Given also prior studies showing synergistic genetic interactions between *Myb*, *jumu*, and *polo* in these same two classes of cardiac progenitor cell divisions^27^, and the regulation of *polo* by both *jumu* and *Myb*^28,41^, our data thus suggests the presence of an additional *CHES-1-like*-independent, *jumu*-regulated pathway involving *Myb*, *polo*, and *neb* that mediates only symmetric and earlier cell divisions. This pathway both expands our prior model of Fox TF-regulated cardiogenic processes (Fig. 8) and emphasizes the central role of *jumu* in heart development by illustrating how it utilizes different combinations of other regulators and downstream effectors to mediate each of these processes.

*neb* encodes a kinesin, the *Drosophila* ortholog of the mammalian Kif14, that is required for chromatin condensation, chromosome segregation, and cytokinesis^31,42–44^; thus errors in one or more of these processes could result in the symmetric or earlier cardiac progenitor cell division defects seen in *neb* mutants. In particular, Neb forms a conserved physical complex with another kinesin, Pavarotti, and the Citron kinase Sticky at the nexus of the central spindle and the actomyosin contractile ring to bring about the cleavage furrow ingression necessary for cytokinesis^45^. Thus it is particularly intriguing to learn from our expression profiling data that both *sticky* (*sti*) and *pavarotti* (*pav*) are also activated by Jumu in the mesoderm [log_2_(fold change) =  − 0.77505; adjusted *P*-value = 8.27 × 10^−8^ and log_2_(fold change) =  − 0.54834; adjusted *P*-value = 2.51 × 10^−6^ respectively in *jumu* mutants] (Supplementary Table S3). These data raise the possibility that Jumu may be mediating one aspect of cardiac progenitor cell divisions by regulating not just one but multiple components of this cytokinesis subnetwork and will be the focus of future investigations.

A particularly interesting question raised by our observations is why loss-of-function mutations in *neb* affect symmetric cell divisions of cardiac progenitor cells, but not asymmetric cell divisions. One can easily conceive of the converse: mutations in *numb* or *pon*, for instance, that disrupt asymmetric cell division without affecting symmetric cell division by failing to appropriately localize Numb to one pole of the dividing cell, and thus disproportionately to one of the resulting daughter cells^27–29^. Proper Numb localization is required only for asymmetric cell division. But why would mutations in *neb*, a gene purportedly involved in more general cell division phenomena such as chromosome condensation, chromosome segregation, and cytokinesis, have no effect on cardiac progenitor asymmetric cell division? One possible explanation could be the presence of other redundant genes and/or pathways that may compensate for the lack of *neb* function during asymmetric cell division. These potential redundant genes and pathways could also be involved in symmetric cell division and may explain why null mutations in *neb* did not result in symmetric cell division defects in each and every hemisegment.

Another intriguing question is whether the activation of *neb* by Jumu is mediated by direct transcriptional regulation, requiring the functional binding of the Fox TF itself to *cis*-regulatory regions of the *neb* gene to stimulate its transcription; or indirect, involving regulation by other, Jumu-activated TFs. While this question cannot be completely answered without the identification and functional analysis of the *cis*-regulatory regions mediating *neb* transcription in the cardiac mesoderm, we note that ChIP-seq analyses compiled in the modERN and modENCODE databases identify three in vivo Jumu binding regions in the immediate neighborhood of the *neb* gene, one overlapping the promoter, the other two in the intron, and all three coinciding with the in vivo binding sites of Myb as well as those of the cardiogenic TFs Tinman, Tailup, and Twist^46,47^ (Supplementary Fig. S2). These observations raise the possibility that one or more of these three Jumu binding sites may be the *cis*-regulatory region(s) mediating *neb* transcription and suggest that *neb* might be a direct transcriptional target of Jumu as well as of Myb.

We have previously suggested^28^ that *FoxM1* is the functional ortholog of *jumu* in mammals because *FoxM1* regulates *Plk1*, the mammalian ortholog of *polo*^48^, because *FoxM1* and *Myb* function as synergistic master regulators of key genetic programs of human B cell proliferation in the germinal center^49^, and because mutations in *FoxM1* exhibit cardiac phenotypes—highly irregular orientation of cardiomyocytes with enlarged polyploid nuclei—that are similar to those in *jumu* mutants^10^. Given our discovery of a *jumu*-regulated pathway for cell division that involves *Myb*, *polo*, and *neb* in *Drosophila*, and possible *cis*-regulatory regions of *neb* that bind both Jumu and Myb, it was very interesting to find that both mammalian FoxM1 and B-Myb bind to the promoter of the *neb* ortholog *Kif14* and activate its transcription in breast cancer cell lines^50,51^. And, not surprisingly, *Kif14* is considered to play a role as an oncogene in cancer types as varied as breast, lung, liver, gastric, colorectal, ovarian, cervical, and prostate cancers in addition to glioma, medulloblastoma, and retinoblastoma^52–64^.

Taken together, these data suggest that the evolutionary role of *neb* and its ortholog *Kif14* is quite crucial, involving not only a conserved complex mediating cytokinesis in both flies and mammals^45,65^, but also its potentially conserved regulation by Fox and Myb TFs. It remains to be seen whether the Fox TF-regulated role of this kinesin is also conserved in cardiac progenitor cell divisions in mammals. The observation that the Meckel–Gruber syndrome linked to some mutations of human *Kif14* is occasionally found to be also associated with congenital heart defects^66^ suggests that this may indeed be the case.

## Methods

### *Drosophila* strains and genetics

The following mutant alleles, deficiencies, and transgenes were used for counting cell division defects: *jumu*^*Df(3R)Exel6157*^ [also known as *Df(3R)Exel6157*; FlyBase ID: FBab0038212] and *CHES-1-like*^*1*^ [also known as *Df(1)CHES-1-like*^*1*^; FlyBase ID: FBab0047384]^28^; *neb*^*k05702*^ [FlyBase ID: FBal0043429]^42^; *neb*^*MI0225*^ [FlyBase ID: FBal0249290]; *UAS-neb*^*RNAi*^ [*neb*^*GD7072*^; FlyBase ID: FBal0210181]; *UAS-neb* [*neb*^*UAS.Tag:HA*^; FlyBase ID: FBal0197607]; *svp-lacZ* [*svp*^*3*^; FlyBase ID: FBal0016610]^33^; *polo*^*10*^ [*polo*^*S132408*^; FlyBase ID: FBal0099891]^67^; *Myb*^*MH107*^ [FlyBase ID: FBal0141652]^68^; *UAS-Dcr-2* [*Dcr-2*^*UAS.cDa*^; FlyBase ID: FBal0211026]^69^; *TinD-GAL4* [*GAL4*^*tin.D*^; FlyBase ID: FBal0267971]^30,39^; and *Hand-GAL4* [*GAL4*^*HCH.Hand*^; FlyBase ID: FBal0190607]^40^. They were maintained over *FM7c, ftz-lacZ*, *CyO, ftz-lacZ*, and *TM3, ftz-lacZ* balancers when they could not be homozygosed before crossing. Relevant embryos resulting from these crosses were distinguished from their siblings by the absence of anti-β-galactosidase staining in the *ftz-lacZ* pattern of the balancers. *CHES-1-like* and *Myb* heterozygotes were distinguished from hemizygotes by both positive staining for anti-Sxl since they were female and the absence of anti-β-galactosidase staining in the *ftz-lacZ* pattern. The same anti-β-galactosidase staining, along with anti-Mef-2 staining, was also used to distinguish the Svp-CCs and Svp-PCs except in the case of RNA interference assays. The *neb*^*MI0225*^ allele, a weak hypomorphic mutation that fails to complement the *neb*^*k05702*^ null allele, was used in genetic interaction assays because it allowed cell division defects to be assessed with increased sensitivity. The genotypes of all the embryos used for counting cell division defects (Supplementary Table S1) and presented in the figures are described below.

**Table:** 

*svp-lacZ/*+	Figure 4a
*neb*^*k05702*^;* svp-lacZ/*+	Figure 4b
*neb*^*MI02225*^*/*+;* svp-lacZ/*+	Figure 5b
*jumu*^*Df(3R)Exel6157*^* svp-lacZ/*+	Figure 5a
*neb*^*MI02225*^*/*+;* jumu*^*Df(3R)Exel6157*^* svp-lacZ/*+	Figure 5c
*polo*^*10*^* svp-lacZ/*+	Figure 5d
*neb*^*MI02225*^*/*+;* polo*^*10*^* svp-lacZ/*+	Figure 5e
*Myb*^*MH107*^*/*+*; svp-lacZ/*+	Figure 5f
*Myb*^*MH107*^*/*+*; neb*^*MI02225*^*/*+;* svp-lacZ/*+	Figure 5g
*CHES-1-like*^*1*^*/*+*; svp-lacZ/*+	Figure 5h
*CHES-1-like*^*1*^*/*+;* neb*^*MI02225*^*/*+;* svp-lacZ/*+	Figure 5i
*jumu*^*Df(3R)Exel6157*^* svp-lacZ/jumu*^*Df(3R)Exel6157*^	Figure 7a
*TinD-GAL4/*+;* jumu*^*Df(3R)Exel6157*^* svp-lacZ/jumu*^*Df(3R)Exel6157*^* UAS-neb*	Figure 7b
+*/*+	Figure 4d
*UAS-neb*^*RNAi*^*/*+	Suppl. Fig. S1
*Hand-GAL4/*+;* TinD-GAL4 UAS-Dcr-2/*+	Suppl. Fig. S1
*Hand-GAL4/*+;* TinD-GAL4 UAS-Dcr-2/UAS-neb*^*RNAi*^	Figure 3e

The following transgenes were used for RT-qPCR: *twi-GAL4* [*GAL4*^*twi.PG*^; FlyBase ID: FBal0040491]^70,71^; *UAS-Dcr-2* [described earlier]; *UAS-jumu*^*RNAi*^ [*jumu*^*GD4099*^; FlyBase ID: FBal0209759]^28,69^; and *UAS-CHES-1-like*^*RNAi*^ [*CHES-1-like*^*GD4327*^; FlyBase ID: FBal0206447]^69,72^. The genotypes of all the embryos used for the RT-qPCR assays are described below:

**Table:** 

*twi-GAL4 UAS-Dcr-2/*+
*twi-GAL4 UAS-Dcr-2/*+;* UAS-jumu*^*RNAi*^*/*+
*twi-GAL4 UAS-Dcr-2/*+;* UAS-CHES-1-like*^*RNAi*^*/*+

### RNA interference assays

RNA interference knockdown of *neb* was performed using the *UAS-neb*^*RNAi*^ (*neb*^*GD7072*^) construct targeted to the cardiac mesoderm using both *TinD-GAL4* and *Hand-GAL4* drivers simultaneously. The efficiency of knockdowns was enhanced with *UAS-Dcr-2*. Cell division defects were counted in the *neb* knockdown and controls (wild-type, *UAS-neb*^*RNAi*^ alone, and both *GAL4* drivers with *UAS-Dcr-2* but no *UAS-neb*^*RNAi*^) by staining with anti-Mef2 and anti-Svp to identify Tin-CCs and Svp-CCs.

### Reverse transcription quantitative real-time PCR (RT-qPCR) assays

Details of the RT-qPCR methodology and the mesoderm-targeted RNA interference knockdown of *jumu* and *CHES-1-like* used in these assays are included in Supplementary Method S1.

### In situ hybridization, immunohistochemistry, microscopy, and cell counting

Embryo fixation, riboprobe synthesis, in situ hybridization, and fluorescent immunohistochemistry were performed as described previously^26,28^. The following primary antibodies were used: rabbit anti-Mef2 (1:1000, gift from B. Paterson; 1:7500, gift from J. Jacobs), mouse anti-β-galactosidase (1:500, Catalog no. Z3783 from Promega), chicken anti-β-galactosidase (1:500, Catalog no. ab9631 from Abcam, Inc), mouse anti-Svp (1:5, monoclonal 5B11 from the Developmental Studies Hybridoma Bank), and mouse anti-Sxl (1:20, monoclonal M18 from the Developmental Studies Hybridoma Bank). Fluorescent microscopy was performed on a Zeiss AxioImager with Apotome. Z-stacks of entire stage 16 embryonic hearts were scanned with a 40X objective and 0.31 µm steps, and all planes for each z-stack were examined to count cells and determine cell division defects. Cell counting and assessment of cell division defects were performed blind (i.e. the individual evaluating cardiac progenitor cell division defects did not know the genotypes of the embryos being assessed) to avoid any potential bias. For all quantitative studies, cells in 179 or more hemisegments were counted for each genotype.

### Statistical methods

Comparison of cell division error rates between genotypes was done using regression models with the response variable being the proportion of hemisegment errors for each embryo. Due to violation of regression assumptions, e.g., non-normality and heteroscedasticity, permutation (randomization) tests were used to obtain reliable *p*-values^73^.

For comparing rates between two genotypes, for example *neb*^*k05702*^ and wild-type, the following general linear model was used:

$${Y}_{j}= {\beta }_{0}+ {\beta }_{1} {I}_{j}+ {\varepsilon }_{j},$$

where ${Y}_{j}$ is the proportion of hemisegment errors for embryo j and indicator variable ${I}_{j}$ is 1 if embryo j has phenotype *neb*^*k05702*^ and 0 otherwise. To obtain a permutation p-value for testing ${H}_{0}: {\beta }_{1}=0$, the estimate of ${\beta }_{1}$ for the actual data is compared with the estimates obtained when the genotypes of the embryos are permuted, i.e., the phenotype labels are randomly shuffled among the embryos in the sample. The permutation test p-value is then $p=(n+1)/(N+1)$ where $n$ is the number of permutation estimates for ${\beta }_{1}$ which exceed the estimate for the actual data and $N$ is the number of permutations^74^. In order to obtain highly reproducible p-values, $N={10}^{6}$ permutations were used for all permutation tests.

For determining if cell division error rates are non-additively related to two gene mutations, for example, to detect synergistic interaction between *neb* and *Myb*, a general linear model allowing for interaction was used:

$${Y}_{j}= {\beta }_{1}{I}_{n,j}+{\beta }_{2}{I}_{M,j}+{\beta }_{3}{I}_{n,j}{I}_{M,j}+{\varepsilon }_{j},$$

where ${I}_{n,j}$ is 1 if the jth embryo is heterozygous for the *neb*^*MI02225*^ mutation and ${I}_{M,j}=1$ if it is heterozygous for the *Myb*^*MH107*^ mutation. Since synergism is present only if ${\beta }_{3}\ne 0$, to detect it ${H}_{0}: {\beta }_{3}=0$ was tested using permutation. Since this is a multiple regression model, a somewhat more sophisticated permutation procedure, the Smith procedure (orthogonalization) was employed^75^.

## Supplementary information

Supplementary Information.

Supplementary Table 1.

Supplementary Table 2.

Supplementary Table 3.

## Data Availability

The Microarray-based expression profiling data used in this study are available from NCBI’S Gene Expression Omnibus through GEO Series accession number GSE65439 (https://www.ncbi.nlm.nih.gov/geo/query/acc.cgi?acc=gse65439). Other data generated and analysed in this study are included in this article and its supplementary information files. All fly strains used in this study are available upon request.
