# Supplementary material for: The Drosophila Forkhead/Fox transcription factor Jumeau mediates specific cardiac progenitor cell divisions by regulating expression of the kinesin Nebbish
Source: Sci Rep. 2021 Feb 5;11:3221. doi: 10.1038/s41598-021-81894-1 (PMC7864957; doi:10.1038/s41598-021-81894-1)
Supplement: Supplementary file 1 — Supplementary Information. [file 41598_2021_81894_MOESM1_ESM.pdf]

The *Drosophila* Forkhead/Fox transcription factor Jumeau mediates specific cardiac progenitor cell divisions by regulating expression of the kinesin Nebbish

Andrew J. Kump[1,2], Manoj Panta[1,2], Kristopher R. Schwab[1,2,3], Mark H. Inlow[2,4],
Shaad M. Ahmad[1,2,3,*]

[1]Department of Biology, Indiana State University, Terre Haute, IN 47809, United States
[2]The Center for Genomic Advocacy, Indiana State University, Terre Haute, IN 47809, United States
[3]The Rich and Robin Porter Cancer Research Center, Indiana State University, Terre Haute, IN 47809, United States
[4]Department of Mathematics and Computer Science, Indiana State University, Terre Haute, IN 47809, United States

*Author for correspondence:

Email: Shaad.Ahmad@indstate.edu

Phone: +1-812-237-2390

Fax: +1-812-237-3378

SUPPLEMENTARY INFORMATION

Supplementary Figures

Figure S1. Cardial cell number defects associated with *neb* knockdown via RNA interference.

Figure S2. Schematic of the *neb* gene showing in vivo binding sites of the cardiogenic transcription factors Jumu, Myb, Tinman, Tailup, and Twist in its immediate vicinity during embryonic stages.

Supplementary Table Legends

Table S1. Quantitative summary and statistical significance of the diverse cell division and positioning defects associated with different genotypes.

Table S2. Quantitative summary and statistical significance of the cardial cell number defects associated with different genotypes in RNA interference assays.

Table S3. Genes activated by *jumu* but not by *CHES-1-like*.

Supplementary Method

Method S1. Assessment of *jumu* and *CHES-1-like* mesoderm-targeted RNAi knockdowns of *neb* transcript expression by reverse transcription quantitative real-time PCR (RT-qPCR).

Supplementary References

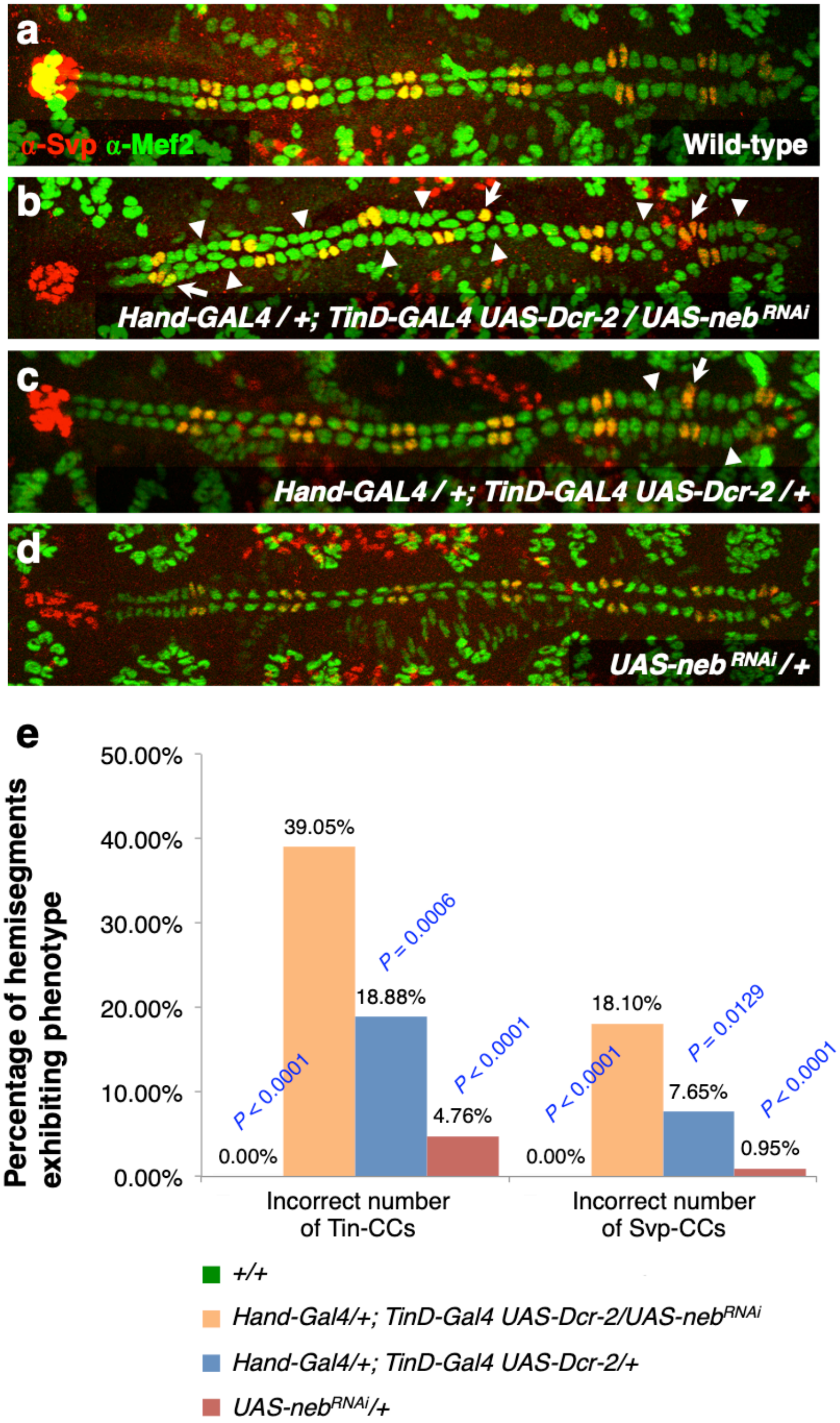

Supplementary Figure S1. Cardial cell number defects associated with *neb* knockdown via RNA interference. (**a**) A wild-type heart stained to show only Tin-CCs (green) and Svp-CCs (yellow). Each hemisegment contains four Tin-CCs and two Svp-CCs. (**b**) Similarly, stained heart from an

embryo where *neb* has been knocked down specifically in the cardiac mesoderm exhibiting changes in Tin-CC numbers and Svp-CC numbers consistent with symmetric (arrowheads) and earlier (arrow) cell division defects, respectively. (**c**) Similarly stained control heart from an embryo possessing one copy each of the cardiac mesoderm-specific *Hand-GAL4* and *TinD-GAL4* drivers but no transgenic *UAS-neb*^{RNAi} constructs. (**d**) Similarly stained control heart from an embryo lacking the cardiac mesoderm-specific *Hand-GAL4* and *TinD-GAL4* drivers but possessing one copy of the transgenic *UAS-neb*^{RNAi} construct. (**e**) Percentage of hemisegments exhibiting Tin-CC and Svp-CC number defects in these genotypes. The statistical significance of each type of cardial cell number defect in the *neb* knockdown compared with the wild-type and control genotypes is shown.

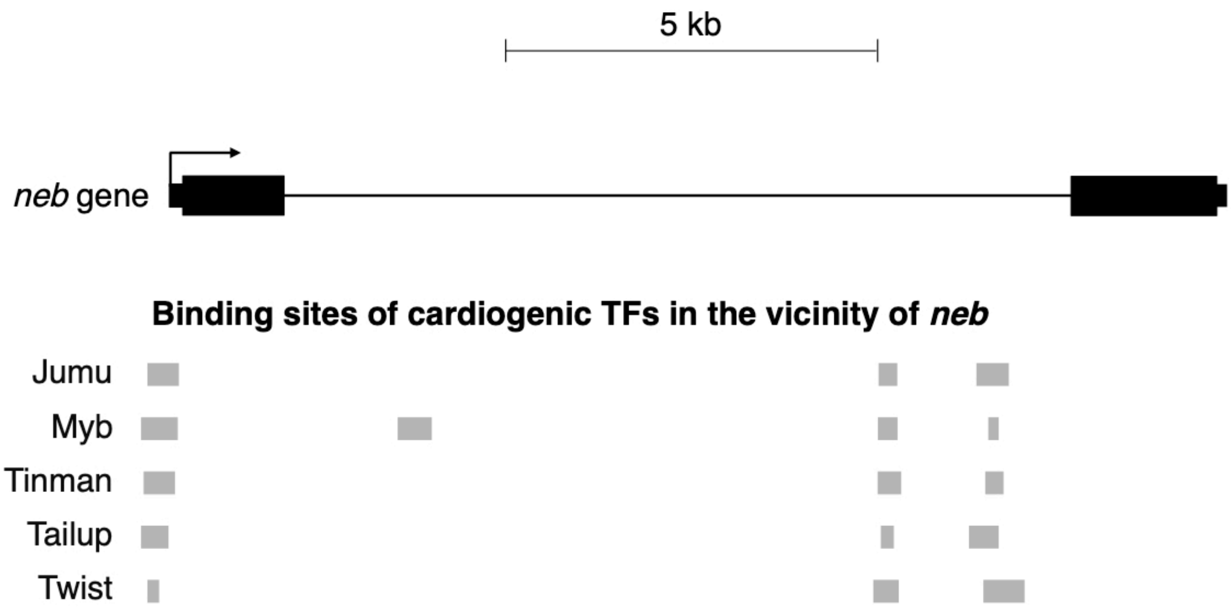

Supplementary Figure S2. Schematic of the *neb* gene showing in vivo binding sites of the cardiogenic transcription factors Jumu, Myb, Tinman, Tailup, and Twist in its immediate vicinity during embryonic stages. The binding data was obtained from ChIP-seq analyses reported in the modERN and modENCODE databases [1-2].

SUPPLEMENTARY TABLE LEGENDS

Supplementary Table S1. Quantitative summary and statistical significance of the diverse cell division and positioning defects associated with different genotypes. (**A**) Detailed quantification of the cell division defects. For each genotype, the denominator in the fraction lists the total number of hemisegments examined, while the numerator reports the number exhibiting the specified defect. All genotypes listed also include one copy of *svp-lacZ*, which has been omitted in the table for the sake of clarity. (**B**) Statistical significance of the cell division defects associated with the genotypes in Table S1A. Individual null hypotheses that were statistically tested using these data are presented.

Supplementary Table S2. Quantitative summary and statistical significance of the cardial cell number defects associated with different genotypes in RNA interference assays. (**A**) Detailed quantification of the cardial cell number defects. For each genotype, the denominator in the fraction lists the total number of hemisegments examined, while the numerator reports the number exhibiting the specified defect. (**B**) Statistical significance of the cardial cell number defects associated with the genotypes in Table S2A. Individual null hypotheses that were statistically tested using these data are presented.

Supplementary Table S3. Genes activated by *jumu* but not by *CHES-1-like*: genes corresponding to probesets exhibiting $\log_2$ Fold Change < -0.5 and adjusted p-value < 0.05 in purified mesodermal cells homozygous for the *jumu* null mutation AND exhibiting $\log_2$ Fold Change ≥ 0 in purified mesodermal cells homozygous for the *CHES-1-like* null mutation.

SUPPLEMENTARY METHOD

Supplementary Method S1. Assessment of *jumu* and *CHES-1-like* mesoderm-targeted RNAi knockdowns of *neb* transcript expression by reverse transcription quantitative real-time PCR (RT-qPCR).

Embryo preparation for total RNA extraction

Stage 11-12 embryos of control and appropriate RNAi knockdown genotypes were collected after being raised at 29°C. Control embryos contained one copy each of the pan-mesodermal *twi-GAL4* driver and *UAS-Dcr-2*, while RNAi knockdown embryos possessed one copy each of the *twi-GAL4* driver and *UAS-Dcr-2* in addition to one copy of the relevant UAS-RNAi construct for *jumu* or *CHES-1-like* (Table 1). Embryos were dechorionated by immersion in 50% bleach for 5 m followed by a quick rinse with 0.1% Triton-X and then with water.

Total RNA isolation and quantification

Total RNA was isolated immediately after the dechorionation and rinse step using the Direct Zol™ RNA MicroPrep Kit (Zymo Research) according to the manufacturer's recommendation which included in-column DNase I treatment to remove genomic DNA. Total RNA was eluted in 10 µl of RNase/DNase-Free water. One µl was used to quantify total RNA concentration and quality using a Thermo Scientific™ NanoDrop™ One Microvolume UV-Vis Spectrophotometer (Table 1).

Embryo	Genotype	Total RNA Concentration	260/280 Ratio	260/230 Ratio
Control	*twi-GAL4 UAS-Dcr-2/+*	0.62 µg/µl	2.16	2.53
jumu RNAi	*twi-GAL4 UAS-Dcr-2/+; jumu*^{GD4099}/+	1.31 µg/µl	2.18	2.46
CHES-1-like RNAi	*twi-GAL4 UAS-Dcr-2/+; CHES-1-like*^{GD4327}/+	0.90 µg/µl	2.16	2.48

Table 1. Genotypes of embryos used as controls and for RNAi knockdowns of specific genes. The concentration and quality of total RNA obtained from these genotypes is listed.

cDNA synthesis for Reverse Transcription quantitative PCR (RT-qPCR)

cDNA was prepared with the SuperScript™ IV VILO™ Master Mix with ezDNase™ Kit (ThermoFisher) using 0.70 µg of total RNA in 20 µl reactions according to the manufacturer's recommendations including the ezDNase™ pre-treatment to remove genomic DNA. The 20 µl cDNA synthesis reactions were diluted 1:5 to a final volume of 100 µl using RNase/DNase-Free water such that they contained the original total RNA at a final concentration of 7.0 ng/µl.

Reverse Transcription quantitative real-time PCR (RT-qPCR)

RT-qPCR was used to quantitate relative transcript expression levels between the different genotypes using the PowerUp™ SYBR™ Green Master Mix (ThermoFisher) in an Applied Biosystems™ QuantStudio™ 3 Real-Time PCR System (ThermoFisher). 10 µl qPCR reactions containing 2 µl (25 ng) of the reverse-transcribed total RNA, 1 pmol each of the relevant forward and reverse primers (Table 2), and 5 µl of the 2X master mix PCR reagent were utilized. qPCR reactions were performed in technical triplicate evaluating C_q reproducibility for each condition that was amplified. The standard deviation of the C_q varied between 0.022 to 0.184 for the technical replicates for each sample, thereby demonstrating C_q reproducibility well below a standard deviation of 0.5 C_q.

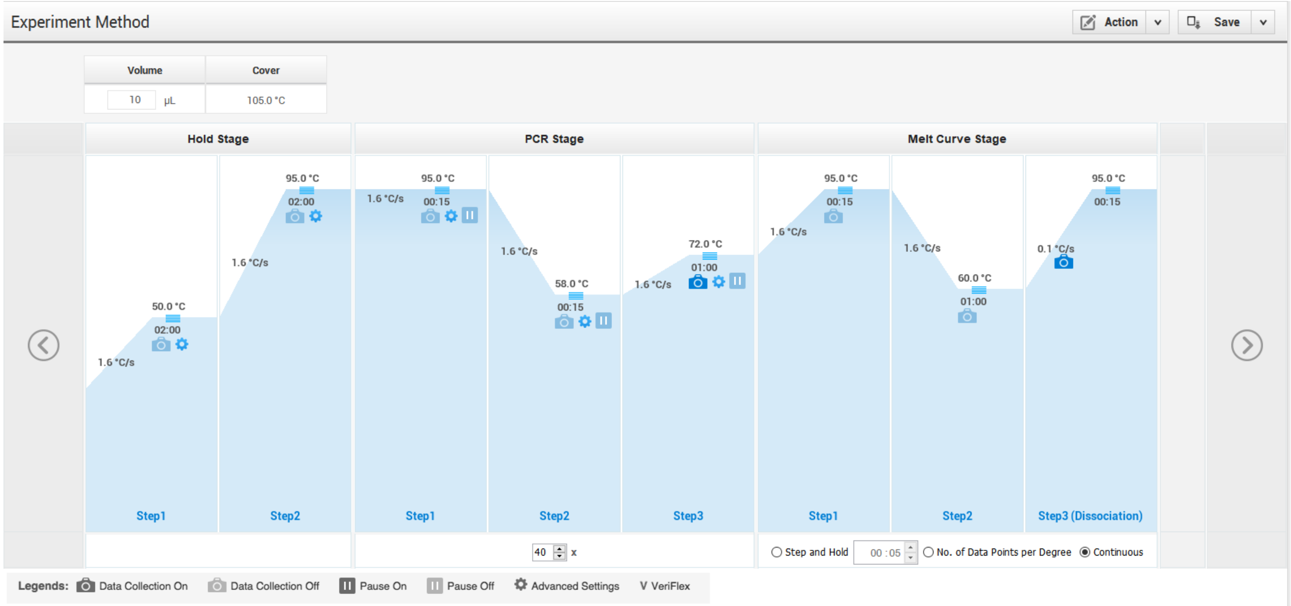

Fig 1. Standard cycling method used for RT-qPCR.

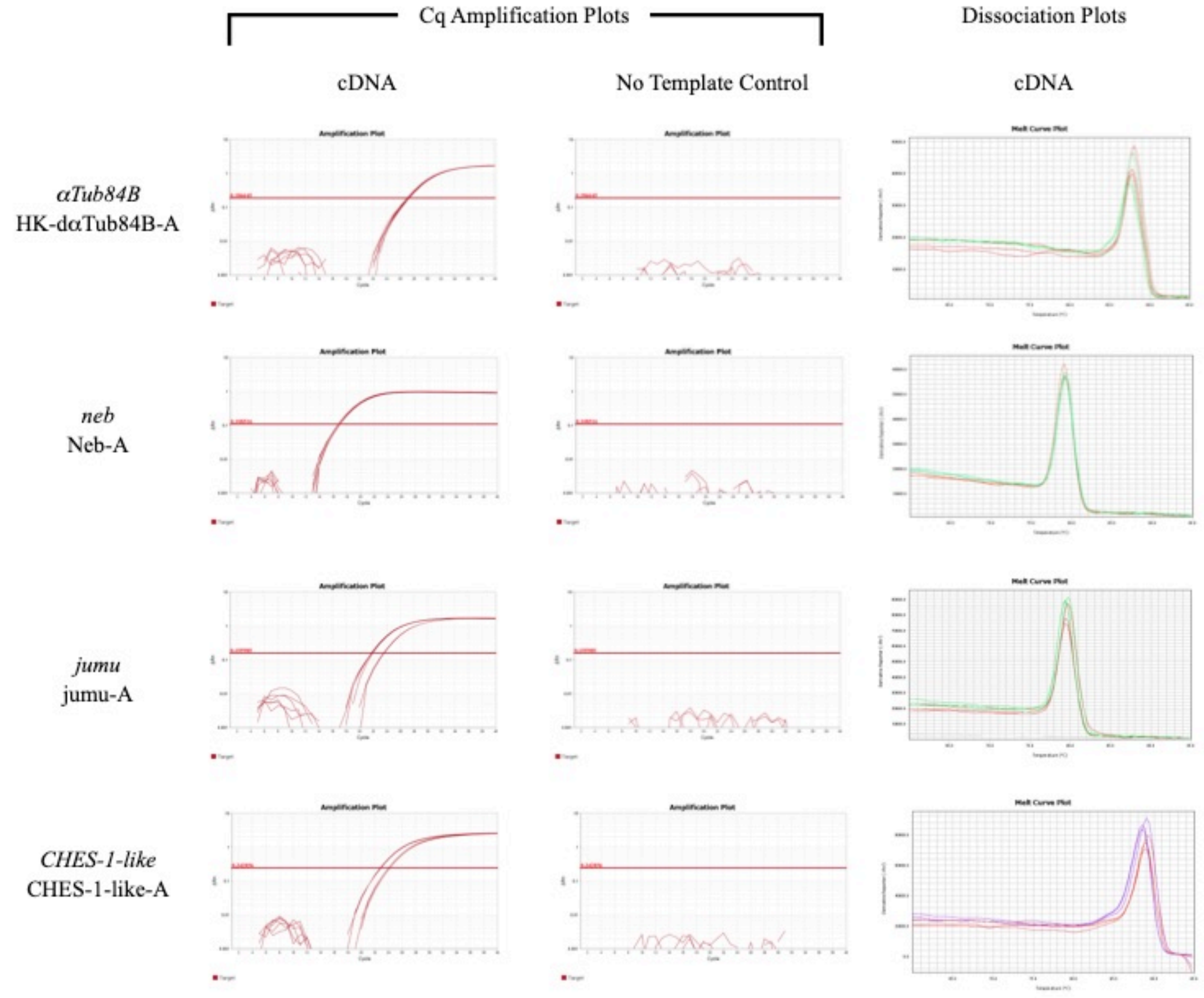

Fig 2. RT-qPCR C_q amplification plots and dissociation plots. Left column: C_q amplification plots for reactions with cDNA obtained by reverse transcription of total RNA for each of the primer sets investigated. Middle column: No template control (NTC) reactions for each primer set. All NTC reactions for each primer set remained below threshold, thereby indicating an absence of amplification and thus no template contamination. Right column: Dissociation (melt curve) plots of each primer set identifies a single amplification product in reactions with cDNA.

Gene	Primer name	Primer sequence 5′ to 3′′	Amplicon size
neb	neb-A-Forward<br>neb-A-Reverse	CAGCACCTTCGTTCCCTATC<br>GCTCGTACTCGTTCCTCAGC	263
jumu	PP16633-Forward<br>PP16633-Reverse	CCTACCCATCAACCATCCAC<br>AATTCGACGCACTGACTCCT	115
CHES-1-like	PP5280-Forward<br>PP5280-Reverse	CCCAGGGTCAGAATAACCAG<br>TGCCGTTGCTGTGATAATGT	180
αTub84B	HK-dαTub84B-A-Forward<br>HK-dαTub84B-A-Reverse	ACGCTCTCTGAGTCAGACCT<br>CCAGCCTGACCAACATGGAT	130

Table 2. Gene-specific primers for RT-qPCR.

QuantStudio™ Design and Analysis Software VERSION 1.4.3 was used to run the experimental method, analyze, and determine the C_q values. The manufacturer's standard cycling method consisting of a UDG activation step, DNA polymerase activation step, PCR amplification stage, and dissociation curve stage was used for all qPCR reactions (Fig 1). The qPCR experimental parameters utilized the standard curve experimental type setting and determined the C_q value using both the Auto-Threshold and Auto-Baseline analysis functions. The qPCR analysis results were exported to Microsoft™ Excel™ for relative gene expression quantification. Relative gene expression was calculated using the $2^{-\Delta\Delta C_T}$ method [3]. *α-Tubulin at 84B* (*αTub84B*) was used as an endogenous reference gene to normalize targets because its primer set demonstrated a low C_q variance between all samples of 27.12 ± 0.18 (Mean C_q ± Standard Deviation) [4, 5].

At least three No Template Control (NTC) qPCR reactions for each primer set were included for each experiment, and they all demonstrated an absence of amplification and thus no template contamination (Fig 2).

SUPPLEMENTARY REFERENCES

1 Kudron, M. M. *et al.* The ModERN Resource: Genome-Wide Binding Profiles for Hundreds of Drosophila and Caenorhabditis elegans Transcription Factors. *Genetics* **208**, 937-949, doi:10.1534/genetics.117.300657 (2018).

2 Negre, N. *et al.* A cis-regulatory map of the Drosophila genome. *Nature* **471**, 527-531, doi:10.1038/nature09990 (2011).

3 Livak, K. J. & Schmittgen, T. D. Analysis of relative gene expression data using real-time quantitative PCR and the 2(-Delta Delta C(T)) Method. *Methods* **25**, 402-408, doi:10.1006/meth.2001.1262 (2001).

4 Ling, D. & Salvaterra, P. M. Robust RT-qPCR data normalization: validation and selection of internal reference genes during post-experimental data analysis. *PLoS One* **6**, e17762, doi:10.1371/journal.pone.0017762 (2011).

5 Bustin, S. A. *et al.* The MIQE guidelines: minimum information for publication of quantitative real-time PCR experiments. *Clinical chemistry* **55**, 611-622, doi:10.1373/clinchem.2008.112797 (2009).
